# Crystal structure of (*E*)-13-{4-[(*Z*)-2-cyano-2-(3,4,5-tri­meth­oxy­phen­yl)ethen­yl]phen­yl}parthenolide methanol hemisolvate

**DOI:** 10.1107/S1600536814019333

**Published:** 2014-09-06

**Authors:** Narsimha Reddy Penthala, Shobanbabu Bommagani, Venumadhav Janganati, Sean Parkin, Peter A. Crooks

**Affiliations:** a Department of Pharmaceutical Sciences, College of Pharmacy, University of Arkansas for Medical Sciences, Little Rock, AR 72205, USA; b Department of Chemistry, University of Kentucky, Lexington KY 40506, USA

**Keywords:** crystal structure, parthenolide derivatives, Heck synthesis, biological activity

## Abstract

The title compound, C_33_H_35_NO_6_ [systematic name: (*Z*)-3-(4-{(*E*)-[(*E*)-1a,5-dimethyl-9-oxo-2,3,7,7a-tetra­hydro­oxireno[2′,3′:9,10]cyclo­deca­[1,2-*b*]furan-8(1a*H*,6*H*,9*H*,10a*H*,10b*H*)-yl­idene]meth­yl}phen­yl)-2-(3,4,5-tri­meth­oxy­phen­yl)acrylo­ni­trile methanol hemisolvate], C_33_H_35_NO_6_·0.5CH_3_OH, was prepared by the reaction of (*Z*)-3-(4-iodo­phen­yl)-2-(3,4,5-tri­meth­oxy­phen­yl)acrylo­nitrile with parthenolide [systematic name: (*E*)-1a,5-dimethyl-8-methyl­ene-2,3,6,7,7a,8,10a,10b-octa­hy­dro­oxireno[2′,3′:9,10]cyclo­deca­[1,2-*b*]furan-9(1a*H*)-one] under Heck reaction conditions. The mol­ecule is built up from fused ten-, five- (lactone) and three-membered (epoxide) rings with a {4-[(*Z*)-2-cyano-2-(3,4,5-tri­meth­oxy­phen­yl)ethen­yl]phen­yl}methyl­idene group as a substituent. The 4-[(*Z*)-2-cyano-2-(3,4,5-tri­meth­oxy­phen­yl)ethen­yl]phenyl group on the parthenolide exocyclic double bond is oriented in a *trans* position to the lactone ring to form the *E* isomer. The dihedral angle between the benzene ring of the phenyl moiety and the lactone ring mean plane is 21.93 (4)°.

## Related literature   

For the biological activity of parthenolide, see: Hall *et al.* (1979 ▶). For the biological activity of parthenolide derivatives similar to the title compound, see: Hanson *et al.* (1970 ▶); Hehner *et al.* (1998 ▶); Kupchan *et al.* (1971 ▶); Neelakantan *et al.* (2009 ▶); Oka *et al.*, 2007 ▶); Ralstin *et al.* (2006 ▶); Sun *et al.* (2006 ▶); Penthala *et al.* (2013*b* ▶). For the synthesis and crystal structures of similar mol­ecules, see: Han *et al.* (2009 ▶); Penthala *et al.* (2013*a* ▶). For details of the experimental procedure, see: Hope (1994 ▶); Parkin & Hope (1998 ▶);
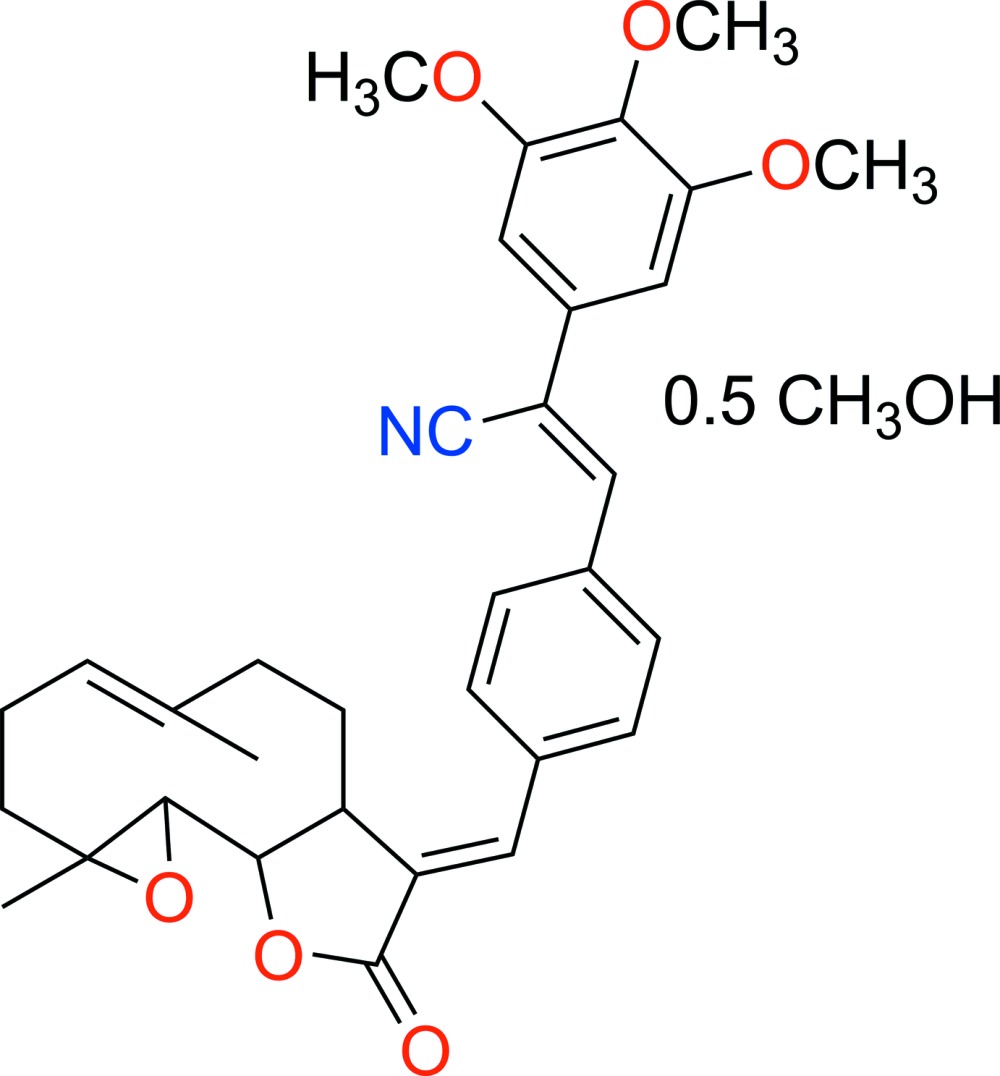


## Experimental   

### Crystal data  

C_33_H_35_NO_6_·0.5CH_4_O
*M* *_r_* = 557.64
Orthorhombic, 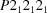

*a* = 9.3347 (2) Å
*b* = 16.2442 (3) Å
*c* = 19.2580 (4) Å
*V* = 2920.18 (10) Å^3^
*Z* = 4
Cu *K*α radiation
μ = 0.71 mm^−1^
*T* = 90 K
0.18 × 0.15 × 0.10 mm

### Data collection  

Bruker X8 Proteum diffractometer
Absorption correction: multi-scan (*SADABS*; Sheldrick, 2008*b* ▶) *T*_min_ = 0.836, *T*_max_ = 0.963
40379 measured reflections
5349 independent reflections
5303 reflections with *I* > 2σ(*I*)
*R*_int_ = 0.036

### Refinement  

*R*[*F* ^2^ > 2σ(*F* ^2^)] = 0.024
*wR*(*F* ^2^) = 0.065
*S* = 1.03
5349 reflections
387 parameters
H-atom parameters constrained
Δρ_max_ = 0.14 e Å^−3^
Δρ_min_ = −0.13 e Å^−3^
Absolute structure: Flack *x* determined using 2283 quotients [(I+)-(I-)]/[(I+)+(I-)] (Parsons *et al.*, 2013 ▶)
Absolute structure parameter: 0.02 (2)

### 

Data collection: *APEX2* (Bruker, 2006 ▶); cell refinement: *APEX2*; data reduction: *APEX2*; program(s) used to solve structure: *SHELXS97* (Sheldrick, 2008*a* ▶); program(s) used to refine structure: *SHELXL2014* (Sheldrick, 2008*a* ▶); molecular graphics: *XP in *SHELXTL** (Sheldrick, 2008*a* ▶); software used to prepare material for publication: *SHELXL97* (Sheldrick, 2008*a* ▶), *CIFFIX* (Parkin, 2013 ▶), *PLATON* (Spek, 2009 ▶) and local program (Parkin, 2000 ▶).

## Supplementary Material

Crystal structure: contains datablock(s) global, I. DOI: 10.1107/S1600536814019333/sj5404sup1.cif

Structure factors: contains datablock(s) I. DOI: 10.1107/S1600536814019333/sj5404Isup2.hkl

**Fig. 1.**
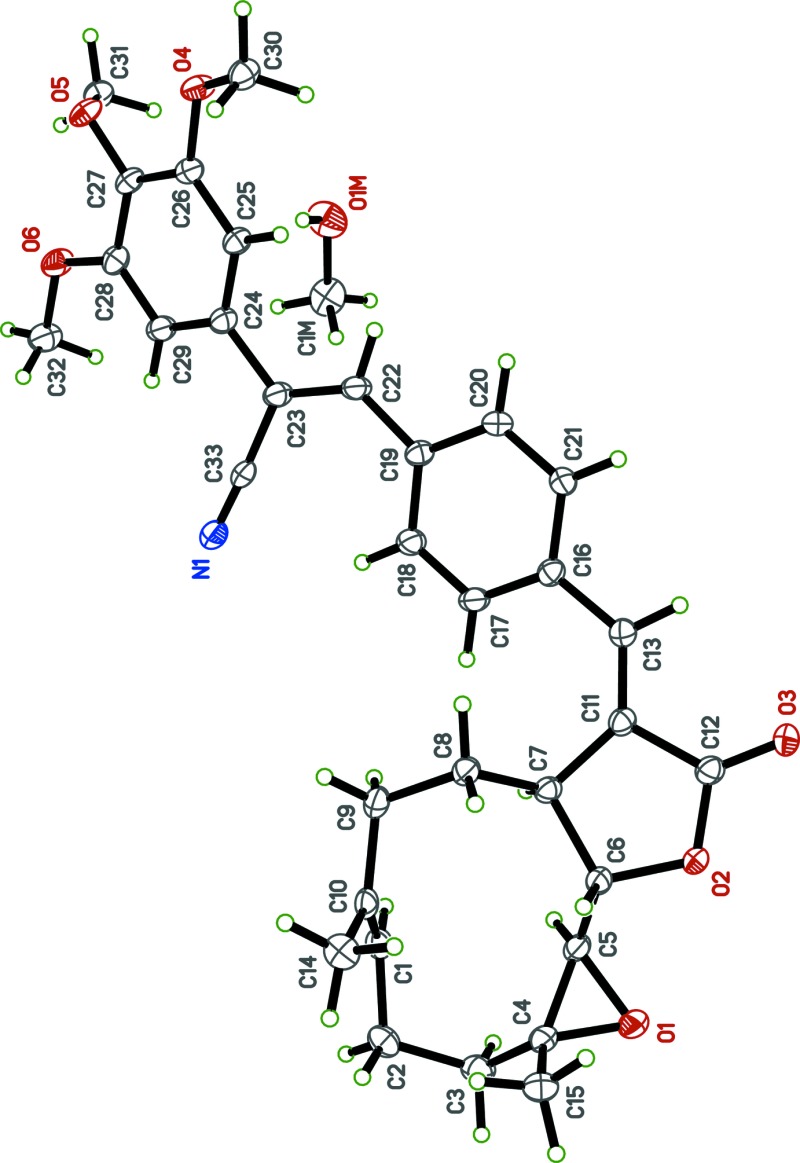
A view of the molecule with displacement ellipsoids drawn at the 50% probability level.

. DOI: 10.1107/S1600536814019333/sj5404fig1.tif
A view of the mol­ecule with displacement ellipsoids drawn at the 50% probability level.

CCDC reference: 1021449

Additional supporting information:  crystallographic information; 3D view; checkCIF report

## supplementary crystallographic information

### S1. Comment

Parthenolide (PTL) and its analogs belong to the class of sesquiterpene lactones. These compounds are currently being used in the development of anti-cancer agents for the treatment of hematological tumours (Sun *et al.*, 2006; Hehner *et al.*, 1998; Ralstin *et al.* 2006; Oka *et al.*, 2007; Kupchan *et al.*, 1971 and Hanson *et al.*, 1970). Recently, we have reported the crystal structure of (*E*)-13-(4-aminophenyl)parthenolide, a Heck reaction derivative of parthenolide (Penthala *et al.* 2013*a*), and we have also reported on *Z*-2-(3,4,5-trimethoxyphenyl)acrylonitrile analogs (Penthala *et al.* 2013*b*) as anti-cancer agents. As part of a program for the development of parthenolide analogs as anti-leukemic agents (Neelakantan *et al.* 2009), and small molecule analogs as anti-cancer agents, our research group is focusing on the synthesis of *E*-olefinic analogues of PTL which can be obtained from the reaction of parthenolide with iodoaromatic reagents utilizing Heck chemistry (Han *et al.* 2009). The title compound was obtained from the reaction of parthenolide with (*Z*)-3-(4-iodophenyl) -2-(3,4,5- trimethoxyphenylacrylonitrile under Heck reaction conditions. To obtain detailed information on the structure of the title compound and to establish the geometry of the exocyclic C13—C14 double bond, a single-crystal X-ray structure determination has been carried out.

Recrystallization of the title compound from methanol afforded light yellow coloured crystals that were suitable for X-ray analysis. The X-ray studies revealed that the title compound was identified as the *E*-isomer (conformation about the exocyclic methylidene C═C bond; the conformation about the C═C bond in the ten-membered ring is also *E*). The molecule is built up from fused ten-, five- (lactone) and three-membered (epoxide) rings with a *(Z)*-3-(4-phenyl)-2-(3,4,5- trimethoxyphenyl)acrylonitrile group as a substituent. The dihedral angle between the benzene ring of the phenyl moiety and the lactone ring mean plane is 21.93 (4) Å.

### S2. Experimental

A mixture of parthenolide (1.0 mmol), diisopropylethylamine (3.0 mmol), and (*Z*)-3-(4-iodophenyl)-2-(3,4,5-trimethoxyphenyl acrylonitrile (1.1 mmol) in toluene (1 ml) was treated with palladium (II) ferrocene (0.01 mmol) and then stirred at 353 K for 24 h. The reaction mixture was cooled to room temperature, water (8 ml) was added, and the mixture was extracted with ethyl acetate (10 mlx3). The separated organics were dried over anhydrous Na_2_SO_4_ and concentrated under reduced pressure. The obtained crude residue was purified using silica flash chromatography (7:3, hexanes/EtOAc) to afford the title compound, which was recrystallized from methanol as light yellow coloured crystals suitable for X-ray analysis (87% yield; M·P.: 478–480 K); ^1^H NMR (400 MHz, CDCl_3_): δ 7.95 (d, *J* = 8.4 Hz, 2H), 7.67 (d, *J* = 3.6 Hz, 1H), 7.52 (d, *J* = 8.4 Hz, 2H), 7.46 (s, 1H), 6.88 (s, 2H), 5.29 (d, *J* = 11.2 Hz, 1H), 3.94 (s, 6H, 2xOCH_3_), 3.90 (s, 3H, OCH_3_), 3.3 (m, 1H), 2.85 (d, *J* = 8.4 Hz, 1H), 2.41–2.46 (m, 1H), 2.10–2.27 (m, 5H), 1.69 (s, 3H, CH_3_), 1.46–1.55 (m, 2H), 1.32 (s, 3H, CH_3_), 1.27–1.30 (m, 1H) *p.p.m.*. ^13^C NMR (100 MHz, CDCl_3_): δ 17.60,17.70, 24.54, 30.58, 36.33, 42.13, 47.16, 55.60, 61.27, 61.94, 66.71, 83.30, 103.75, 113.21, 118.04, 125.48, 129.53, 129.95, 130.56, 130.97, 134.88, 134.96, 135.69, 137.06, 139.77, 140.34, 153.89, 170.85 *p.p.m.*.

### S3. Refinement

Crystal data, data collection and structure refinement details are summarized in Table 1. H atoms were found in difference Fourier maps, but subsequently included in the refinement using riding models, with constrained distances set to 0.95%A (C_sp2_H), 0.98Å (RCH_3_), 0.99Å (*R*_2_CH_2_), 1.00Å (*R*_3_CH) and 0.84Å (OH). *U*_iso_(H) parameters were set to values of either 1.2*U*_eq_ or 1.5*U*_eq_ (RCH_3_ and OH only) of the attached atom.

The partial occupancy methanol molecule refined to an occupancy of about one half. For the final rounds of refinement its occupancy was fixed at exactly 0.5 for the sake of simplicity. This is reasonable because other crystals from the same batch would almost certainly have had varying amounts of solvent incorporated, due to unpredictable rates of solvent loss dependent on such things as crystal handling. The position of this half-occupancy methanol is consistent with an O—H···π weak hydrogen bonding interaction in which the distance between atom O1M and the centroid of the trimethoxyphenyl ring (C24-C29) is 3.212 (3)Å.

### Crystal data

**Table:**

C_33_H_35_NO_6_·0.5CH_4_O	*D*_x_ = 1.268 Mg m^−^^3^
*M**_r_* = 557.64	Cu *K*α radiation, λ = 1.54178 Å
Orthorhombic, *P*2_1_2_1_2_1_	Cell parameters from 9693 reflections
*a* = 9.3347 (2) Å	θ = 3.6–68.4°
*b* = 16.2442 (3) Å	µ = 0.71 mm^−^^1^
*c* = 19.2580 (4) Å	*T* = 90 K
*V* = 2920.18 (10) Å^3^	Irregular cut wedge, pale yellow
*Z* = 4	0.18 × 0.15 × 0.10 mm
*F*(000) = 1188	

### Data collection

**Table:**

Bruker X8 Proteum diffractometer	5349 independent reflections
Radiation source: fine-focus rotating anode	5303 reflections with *I* > 2σ(*I*)
Detector resolution: 5.6 pixels mm^-1^	*R*_int_ = 0.036
φ and ω scans	θ_max_ = 68.4°, θ_min_ = 3.6°
Absorption correction: multi-scan (*SADABS*; Sheldrick, 2008*b*)	*h* = −11→11
*T*_min_ = 0.836, *T*_max_ = 0.963	*k* = −13→19
40379 measured reflections	*l* = −20→23

### Refinement

**Table:**

Refinement on *F*^2^	Hydrogen site location: difference Fourier map
Least-squares matrix: full	H-atom parameters constrained
*R*[*F*^2^ > 2σ(*F*^2^)] = 0.024	*w* = 1/[σ^2^(*F*_o_^2^) + (0.0363*P*)^2^ + 0.5907*P*] where *P* = (*F*_o_^2^ + 2*F*_c_^2^)/3
*wR*(*F*^2^) = 0.065	(Δ/σ)_max_ < 0.001
*S* = 1.03	Δρ_max_ = 0.14 e Å^−^^3^
5349 reflections	Δρ_min_ = −0.13 e Å^−^^3^
387 parameters	Extinction correction: *SHELXL2014* (Sheldrick, 2008a), Fc^*^=kFc[1+0.001xFc^2^λ^3^/sin(2θ)]^-1/4^
0 restraints	Extinction coefficient: 0.00092 (14)
Primary atom site location: structure-invariant direct methods	Absolute structure: Flack *x* determined using 2283 quotients [(I+)-(I-)]/[(I+)+(I-)] (Parsons *et al.*, 2013)
Secondary atom site location: difference Fourier map	Absolute structure parameter: 0.02 (2)

### Special details

**Table:**

Experimental. The crystal was mounted with polyisobutene oil on the tip of a fine glass fibre, which was fastened in a copper mounting pin with electrical solder. It was placed directly into the cold gas stream of a liquid nitrogen based cryostat, according to published methods (Hope, 1994; Parkin & Hope, 1998).Diffraction data were collected with the crystal at 90 K, which is standard practice in this laboratory for the majority of flash-cooled crystals.
Geometry. All e.s.d.'s (except the e.s.d. in the dihedral angle between two l.s. planes) are estimated using the full covariance matrix. The cell e.s.d.'s are taken into account individually in the estimation of e.s.d.'s in distances, angles and torsion angles; correlations between e.s.d.'s in cell parameters are only used when they are defined by crystal symmetry. An approximate (isotropic) treatment of cell e.s.d.'s is used for estimating e.s.d.'s involving l.s. planes.
Refinement. Refinement progress was checked using *PLATON* (Spek, 2009) and by an *R*-tensor (Parkin, 2000). The final model was further checked with the IUCr utility *checkCIF*.

### Fractional atomic coordinates and isotropic or equivalent isotropic displacement parameters (Å^2^)

**Table:**

	*x*	*y*	*z*	*U*_iso_*/*U*_eq_	Occ. (<1)
O1	0.48351 (13)	0.18048 (7)	0.54248 (6)	0.0242 (3)	
O2	0.35142 (13)	0.32648 (7)	0.48438 (5)	0.0210 (2)	
O3	0.34451 (13)	0.45934 (7)	0.45520 (6)	0.0237 (3)	
O4	0.02191 (12)	0.41510 (7)	−0.22367 (6)	0.0225 (3)	
O5	0.14060 (12)	0.30869 (7)	−0.31036 (5)	0.0219 (2)	
O6	0.30389 (14)	0.18270 (7)	−0.26366 (6)	0.0261 (3)	
N1	0.35753 (19)	0.14312 (9)	0.01607 (8)	0.0311 (4)	
C1	0.35744 (17)	0.04178 (10)	0.37932 (8)	0.0203 (3)	
H1	0.4318	0.0590	0.3489	0.024*	
C2	0.40090 (19)	−0.01340 (10)	0.43865 (9)	0.0231 (3)	
H2A	0.4617	−0.0586	0.4208	0.028*	
H2B	0.3143	−0.0381	0.4598	0.028*	
C3	0.48435 (19)	0.03537 (10)	0.49458 (9)	0.0249 (4)	
H3A	0.5000	0.0001	0.5358	0.030*	
H3B	0.5792	0.0516	0.4761	0.030*	
C4	0.40199 (18)	0.11124 (10)	0.51545 (8)	0.0213 (3)	
C5	0.42653 (17)	0.18504 (10)	0.47296 (8)	0.0187 (3)	
H5	0.5005	0.1772	0.4360	0.022*	
C6	0.31309 (17)	0.24713 (9)	0.45417 (8)	0.0180 (3)	
H6	0.2182	0.2288	0.4727	0.022*	
C7	0.30239 (17)	0.26039 (9)	0.37424 (8)	0.0170 (3)	
H7	0.3909	0.2376	0.3522	0.020*	
C8	0.17085 (17)	0.21982 (10)	0.33974 (8)	0.0197 (3)	
H8A	0.0932	0.2152	0.3745	0.024*	
H8B	0.1362	0.2559	0.3019	0.024*	
C9	0.20236 (18)	0.13380 (10)	0.30982 (8)	0.0211 (3)	
H9A	0.1211	0.1168	0.2801	0.025*	
H9B	0.2887	0.1370	0.2801	0.025*	
C10	0.22602 (17)	0.06913 (9)	0.36481 (8)	0.0189 (3)	
C11	0.30617 (16)	0.35309 (9)	0.36819 (8)	0.0175 (3)	
C12	0.33264 (17)	0.38809 (9)	0.43821 (8)	0.0191 (3)	
C13	0.29827 (17)	0.40414 (9)	0.31380 (8)	0.0188 (3)	
H13	0.2959	0.4610	0.3254	0.023*	
C14	0.09048 (18)	0.04115 (11)	0.39995 (9)	0.0253 (4)	
H14A	0.1121	−0.0048	0.4313	0.038*	
H14B	0.0213	0.0231	0.3648	0.038*	
H14C	0.0498	0.0869	0.4267	0.038*	
C15	0.2653 (2)	0.09726 (11)	0.55515 (9)	0.0265 (4)	
H15A	0.2874	0.0715	0.5999	0.040*	
H15B	0.2021	0.0610	0.5283	0.040*	
H15C	0.2175	0.1501	0.5631	0.040*	
C16	0.29273 (16)	0.38648 (9)	0.23919 (8)	0.0175 (3)	
C17	0.33360 (19)	0.31137 (10)	0.20977 (8)	0.0217 (3)	
H17	0.3699	0.2690	0.2389	0.026*	
C18	0.32232 (19)	0.29741 (10)	0.13921 (8)	0.0226 (3)	
H18	0.3514	0.2459	0.1207	0.027*	
C19	0.26861 (17)	0.35823 (10)	0.09458 (8)	0.0182 (3)	
C20	0.23537 (17)	0.43490 (10)	0.12358 (8)	0.0189 (3)	
H20	0.2037	0.4782	0.0942	0.023*	
C21	0.24764 (17)	0.44897 (9)	0.19415 (8)	0.0191 (3)	
H21	0.2252	0.5018	0.2123	0.023*	
C22	0.24427 (18)	0.34952 (10)	0.01995 (8)	0.0202 (3)	
H22	0.2131	0.3986	−0.0022	0.024*	
C23	0.25803 (18)	0.28451 (10)	−0.02315 (8)	0.0201 (3)	
C24	0.22173 (17)	0.28790 (10)	−0.09850 (8)	0.0201 (3)	
C25	0.13268 (17)	0.35015 (10)	−0.12349 (8)	0.0194 (3)	
H25	0.0903	0.3882	−0.0922	0.023*	
C26	0.10621 (17)	0.35628 (10)	−0.19439 (8)	0.0188 (3)	
C27	0.16914 (17)	0.30063 (10)	−0.24085 (8)	0.0189 (3)	
C28	0.25283 (18)	0.23611 (10)	−0.21509 (8)	0.0213 (3)	
C29	0.27962 (18)	0.22993 (10)	−0.14397 (8)	0.0229 (3)	
H29	0.3372	0.1863	−0.1266	0.027*	
C30	−0.07334 (18)	0.45919 (11)	−0.17877 (8)	0.0228 (3)	
H30A	−0.1350	0.4201	−0.1541	0.034*	
H30B	−0.1327	0.4966	−0.2064	0.034*	
H30C	−0.0175	0.4910	−0.1451	0.034*	
C31	0.26452 (18)	0.32355 (10)	−0.35293 (8)	0.0219 (3)	
H31A	0.3352	0.3552	−0.3265	0.033*	
H31B	0.2362	0.3547	−0.3943	0.033*	
H31C	0.3065	0.2709	−0.3671	0.033*	
C32	0.3951 (2)	0.11810 (11)	−0.24020 (9)	0.0294 (4)	
H32A	0.4758	0.1416	−0.2143	0.044*	
H32B	0.4314	0.0873	−0.2802	0.044*	
H32C	0.3409	0.0810	−0.2099	0.044*	
C33	0.31313 (19)	0.20649 (10)	0.00030 (8)	0.0219 (3)	
O1M	0.4544 (3)	0.42249 (17)	−0.16592 (16)	0.0371 (6)	0.5
H1M	0.3705	0.4054	−0.1596	0.056*	0.5
C1M	0.5516 (4)	0.3704 (3)	−0.1313 (2)	0.0338 (8)	0.5
H1M1	0.6414	0.4001	−0.1230	0.051*	0.5
H1M2	0.5705	0.3218	−0.1600	0.051*	0.5
H1M3	0.5104	0.3532	−0.0868	0.051*	0.5

### Atomic displacement parameters (Å^2^)

**Table:**

	*U*^11^	*U*^22^	*U*^33^	*U*^12^	*U*^13^	*U*^23^
O1	0.0286 (6)	0.0255 (6)	0.0187 (5)	−0.0042 (5)	−0.0075 (5)	0.0020 (5)
O2	0.0323 (6)	0.0182 (5)	0.0125 (5)	−0.0012 (5)	−0.0003 (5)	−0.0012 (4)
O3	0.0345 (7)	0.0187 (5)	0.0181 (5)	−0.0004 (5)	−0.0002 (5)	−0.0042 (4)
O4	0.0247 (6)	0.0275 (6)	0.0153 (5)	0.0082 (5)	−0.0013 (5)	0.0017 (5)
O5	0.0190 (6)	0.0349 (6)	0.0118 (5)	0.0016 (5)	−0.0008 (4)	0.0001 (5)
O6	0.0331 (7)	0.0291 (6)	0.0161 (5)	0.0110 (5)	−0.0011 (5)	−0.0044 (5)
N1	0.0477 (10)	0.0227 (7)	0.0228 (7)	0.0045 (7)	−0.0131 (7)	−0.0036 (6)
C1	0.0219 (8)	0.0184 (7)	0.0205 (8)	−0.0015 (6)	0.0034 (6)	−0.0030 (6)
C2	0.0208 (8)	0.0193 (7)	0.0293 (9)	0.0014 (6)	0.0023 (7)	0.0014 (7)
C3	0.0251 (8)	0.0239 (8)	0.0258 (8)	0.0009 (7)	−0.0036 (7)	0.0060 (7)
C4	0.0245 (8)	0.0228 (8)	0.0165 (7)	−0.0033 (7)	−0.0049 (7)	0.0017 (6)
C5	0.0200 (8)	0.0223 (8)	0.0137 (7)	−0.0030 (6)	−0.0017 (6)	−0.0007 (6)
C6	0.0217 (8)	0.0178 (7)	0.0145 (7)	−0.0029 (6)	0.0008 (6)	−0.0011 (6)
C7	0.0189 (7)	0.0183 (7)	0.0137 (7)	0.0018 (6)	0.0008 (6)	−0.0001 (6)
C8	0.0226 (8)	0.0192 (7)	0.0172 (7)	0.0022 (6)	−0.0032 (6)	−0.0011 (6)
C9	0.0252 (8)	0.0208 (8)	0.0174 (7)	−0.0003 (6)	−0.0019 (6)	−0.0049 (6)
C10	0.0232 (8)	0.0165 (7)	0.0169 (7)	−0.0015 (6)	0.0018 (6)	−0.0057 (6)
C11	0.0179 (7)	0.0192 (7)	0.0154 (7)	0.0020 (6)	0.0005 (6)	−0.0017 (6)
C12	0.0209 (8)	0.0205 (8)	0.0158 (7)	0.0009 (6)	0.0022 (6)	0.0000 (6)
C13	0.0206 (7)	0.0175 (7)	0.0183 (7)	0.0008 (6)	−0.0012 (6)	−0.0012 (6)
C14	0.0209 (8)	0.0256 (8)	0.0295 (9)	0.0005 (7)	0.0010 (7)	0.0009 (7)
C15	0.0329 (9)	0.0265 (8)	0.0201 (8)	−0.0039 (7)	0.0032 (7)	0.0039 (7)
C16	0.0160 (7)	0.0200 (7)	0.0165 (7)	−0.0013 (6)	−0.0008 (6)	0.0002 (6)
C17	0.0293 (9)	0.0201 (7)	0.0158 (7)	0.0052 (7)	−0.0011 (6)	0.0029 (6)
C18	0.0316 (9)	0.0192 (8)	0.0172 (7)	0.0065 (7)	−0.0005 (7)	−0.0003 (6)
C19	0.0194 (7)	0.0205 (7)	0.0147 (7)	0.0005 (6)	0.0011 (6)	0.0015 (6)
C20	0.0196 (7)	0.0192 (7)	0.0178 (7)	0.0018 (6)	−0.0006 (6)	0.0040 (6)
C21	0.0226 (8)	0.0164 (7)	0.0183 (7)	0.0002 (6)	0.0002 (6)	−0.0005 (6)
C22	0.0245 (8)	0.0202 (7)	0.0159 (7)	0.0027 (7)	−0.0007 (6)	0.0042 (6)
C23	0.0226 (8)	0.0219 (8)	0.0158 (7)	0.0015 (7)	−0.0013 (6)	0.0023 (6)
C24	0.0237 (8)	0.0215 (7)	0.0151 (7)	−0.0016 (6)	−0.0008 (6)	0.0014 (6)
C25	0.0215 (8)	0.0227 (7)	0.0140 (7)	0.0003 (6)	0.0002 (6)	−0.0006 (6)
C26	0.0177 (7)	0.0217 (7)	0.0170 (7)	−0.0006 (6)	−0.0011 (6)	0.0024 (6)
C27	0.0174 (7)	0.0263 (8)	0.0130 (7)	−0.0016 (6)	−0.0006 (6)	0.0010 (6)
C28	0.0225 (8)	0.0245 (8)	0.0168 (7)	0.0003 (7)	0.0003 (6)	−0.0029 (6)
C29	0.0277 (8)	0.0229 (8)	0.0181 (8)	0.0047 (7)	−0.0028 (6)	0.0007 (6)
C30	0.0222 (8)	0.0269 (8)	0.0192 (8)	0.0048 (7)	−0.0010 (6)	−0.0031 (7)
C31	0.0230 (8)	0.0264 (8)	0.0165 (7)	0.0005 (7)	0.0029 (6)	0.0007 (6)
C32	0.0359 (10)	0.0292 (9)	0.0231 (8)	0.0125 (8)	−0.0005 (8)	−0.0024 (7)
C33	0.0301 (8)	0.0225 (8)	0.0130 (7)	−0.0001 (7)	−0.0046 (6)	−0.0031 (6)
O1M	0.0289 (13)	0.0360 (14)	0.0465 (16)	−0.0027 (12)	0.0055 (12)	0.0064 (13)
C1M	0.0213 (17)	0.044 (2)	0.0359 (19)	−0.0039 (15)	0.0058 (16)	−0.0010 (18)

### Geometric parameters (Å, º)

**Table:**

O1—C5	1.4426 (18)	C14—H14B	0.9800
O1—C4	1.454 (2)	C14—H14C	0.9800
O2—C12	1.3500 (19)	C15—H15A	0.9800
O2—C6	1.4587 (18)	C15—H15B	0.9800
O3—C12	1.208 (2)	C15—H15C	0.9800
O4—C26	1.3602 (19)	C16—C17	1.398 (2)
O4—C30	1.4321 (19)	C16—C21	1.400 (2)
O5—C27	1.3713 (18)	C17—C18	1.382 (2)
O5—C31	1.4381 (19)	C17—H17	0.9500
O6—C28	1.3619 (19)	C18—C19	1.402 (2)
O6—C32	1.425 (2)	C18—H18	0.9500
N1—C33	1.151 (2)	C19—C20	1.400 (2)
C1—C10	1.334 (2)	C19—C22	1.462 (2)
C1—C2	1.508 (2)	C20—C21	1.383 (2)
C1—H1	0.9500	C20—H20	0.9500
C2—C3	1.547 (2)	C21—H21	0.9500
C2—H2A	0.9900	C22—C23	1.349 (2)
C2—H2B	0.9900	C22—H22	0.9500
C3—C4	1.507 (2)	C23—C33	1.440 (2)
C3—H3A	0.9900	C23—C24	1.491 (2)
C3—H3B	0.9900	C24—C29	1.395 (2)
C4—C5	1.470 (2)	C24—C25	1.395 (2)
C4—C15	1.505 (2)	C25—C26	1.391 (2)
C5—C6	1.506 (2)	C25—H25	0.9500
C5—H5	1.0000	C26—C27	1.401 (2)
C6—C7	1.558 (2)	C27—C28	1.398 (2)
C6—H6	1.0000	C28—C29	1.396 (2)
C7—C11	1.511 (2)	C29—H29	0.9500
C7—C8	1.544 (2)	C30—H30A	0.9800
C7—H7	1.0000	C30—H30B	0.9800
C8—C9	1.540 (2)	C30—H30C	0.9800
C8—H8A	0.9900	C31—H31A	0.9800
C8—H8B	0.9900	C31—H31B	0.9800
C9—C10	1.508 (2)	C31—H31C	0.9800
C9—H9A	0.9900	C32—H32A	0.9800
C9—H9B	0.9900	C32—H32B	0.9800
C10—C14	1.505 (2)	C32—H32C	0.9800
C11—C13	1.338 (2)	O1M—C1M	1.408 (5)
C11—C12	1.484 (2)	O1M—H1M	0.8400
C13—C16	1.466 (2)	C1M—H1M1	0.9800
C13—H13	0.9500	C1M—H1M2	0.9800
C14—H14A	0.9800	C1M—H1M3	0.9800
			
C5—O1—C4	60.96 (10)	C4—C15—H15A	109.5
C12—O2—C6	111.14 (11)	C4—C15—H15B	109.5
C26—O4—C30	117.40 (12)	H15A—C15—H15B	109.5
C27—O5—C31	114.60 (12)	C4—C15—H15C	109.5
C28—O6—C32	117.42 (12)	H15A—C15—H15C	109.5
C10—C1—C2	127.16 (15)	H15B—C15—H15C	109.5
C10—C1—H1	116.4	C17—C16—C21	117.62 (14)
C2—C1—H1	116.4	C17—C16—C13	123.93 (14)
C1—C2—C3	111.01 (13)	C21—C16—C13	118.42 (14)
C1—C2—H2A	109.4	C18—C17—C16	121.39 (15)
C3—C2—H2A	109.4	C18—C17—H17	119.3
C1—C2—H2B	109.4	C16—C17—H17	119.3
C3—C2—H2B	109.4	C17—C18—C19	120.96 (14)
H2A—C2—H2B	108.0	C17—C18—H18	119.5
C4—C3—C2	110.34 (14)	C19—C18—H18	119.5
C4—C3—H3A	109.6	C20—C19—C18	117.49 (14)
C2—C3—H3A	109.6	C20—C19—C22	116.35 (14)
C4—C3—H3B	109.6	C18—C19—C22	126.16 (15)
C2—C3—H3B	109.6	C21—C20—C19	121.39 (14)
H3A—C3—H3B	108.1	C21—C20—H20	119.3
O1—C4—C5	59.12 (10)	C19—C20—H20	119.3
O1—C4—C15	112.24 (13)	C20—C21—C16	120.93 (14)
C5—C4—C15	122.56 (15)	C20—C21—H21	119.5
O1—C4—C3	117.45 (14)	C16—C21—H21	119.5
C5—C4—C3	116.05 (14)	C23—C22—C19	131.73 (15)
C15—C4—C3	116.40 (14)	C23—C22—H22	114.1
O1—C5—C4	59.92 (10)	C19—C22—H22	114.1
O1—C5—C6	121.09 (13)	C22—C23—C33	122.00 (14)
C4—C5—C6	124.78 (14)	C22—C23—C24	123.24 (15)
O1—C5—H5	113.6	C33—C23—C24	114.74 (14)
C4—C5—H5	113.6	C29—C24—C25	120.25 (14)
C6—C5—H5	113.6	C29—C24—C23	119.87 (14)
O2—C6—C5	108.87 (12)	C25—C24—C23	119.86 (14)
O2—C6—C7	106.71 (12)	C26—C25—C24	119.76 (15)
C5—C6—C7	112.02 (12)	C26—C25—H25	120.1
O2—C6—H6	109.7	C24—C25—H25	120.1
C5—C6—H6	109.7	O4—C26—C25	124.05 (14)
C7—C6—H6	109.7	O4—C26—C27	115.54 (13)
C11—C7—C8	114.26 (13)	C25—C26—C27	120.41 (15)
C11—C7—C6	102.28 (12)	O5—C27—C28	121.77 (14)
C8—C7—C6	114.66 (13)	O5—C27—C26	118.71 (14)
C11—C7—H7	108.4	C28—C27—C26	119.43 (14)
C8—C7—H7	108.4	O6—C28—C29	124.43 (15)
C6—C7—H7	108.4	O6—C28—C27	115.43 (13)
C9—C8—C7	113.36 (13)	C29—C28—C27	120.14 (14)
C9—C8—H8A	108.9	C24—C29—C28	119.87 (15)
C7—C8—H8A	108.9	C24—C29—H29	120.1
C9—C8—H8B	108.9	C28—C29—H29	120.1
C7—C8—H8B	108.9	O4—C30—H30A	109.5
H8A—C8—H8B	107.7	O4—C30—H30B	109.5
C10—C9—C8	113.42 (12)	H30A—C30—H30B	109.5
C10—C9—H9A	108.9	O4—C30—H30C	109.5
C8—C9—H9A	108.9	H30A—C30—H30C	109.5
C10—C9—H9B	108.9	H30B—C30—H30C	109.5
C8—C9—H9B	108.9	O5—C31—H31A	109.5
H9A—C9—H9B	107.7	O5—C31—H31B	109.5
C1—C10—C14	125.32 (15)	H31A—C31—H31B	109.5
C1—C10—C9	120.92 (15)	O5—C31—H31C	109.5
C14—C10—C9	113.76 (14)	H31A—C31—H31C	109.5
C13—C11—C12	118.88 (14)	H31B—C31—H31C	109.5
C13—C11—C7	132.60 (14)	O6—C32—H32A	109.5
C12—C11—C7	108.40 (13)	O6—C32—H32B	109.5
O3—C12—O2	121.34 (14)	H32A—C32—H32B	109.5
O3—C12—C11	128.93 (15)	O6—C32—H32C	109.5
O2—C12—C11	109.63 (13)	H32A—C32—H32C	109.5
C11—C13—C16	130.38 (15)	H32B—C32—H32C	109.5
C11—C13—H13	114.8	N1—C33—C23	177.04 (16)
C16—C13—H13	114.8	C1M—O1M—H1M	109.5
C10—C14—H14A	109.5	O1M—C1M—H1M1	109.5
C10—C14—H14B	109.5	O1M—C1M—H1M2	109.5
H14A—C14—H14B	109.5	H1M1—C1M—H1M2	109.5
C10—C14—H14C	109.5	O1M—C1M—H1M3	109.5
H14A—C14—H14C	109.5	H1M1—C1M—H1M3	109.5
H14B—C14—H14C	109.5	H1M2—C1M—H1M3	109.5
			
C10—C1—C2—C3	−107.33 (19)	C11—C13—C16—C17	−18.2 (3)
C1—C2—C3—C4	51.42 (18)	C11—C13—C16—C21	163.60 (17)
C5—O1—C4—C15	−115.66 (16)	C21—C16—C17—C18	−3.6 (2)
C5—O1—C4—C3	105.42 (16)	C13—C16—C17—C18	178.17 (16)
C2—C3—C4—O1	−154.06 (13)	C16—C17—C18—C19	−0.3 (3)
C2—C3—C4—C5	−87.00 (17)	C17—C18—C19—C20	3.8 (2)
C2—C3—C4—C15	68.71 (18)	C17—C18—C19—C22	−176.49 (17)
C4—O1—C5—C6	114.86 (17)	C18—C19—C20—C21	−3.4 (2)
C15—C4—C5—O1	98.13 (16)	C22—C19—C20—C21	176.89 (15)
C3—C4—C5—O1	−107.79 (15)	C19—C20—C21—C16	−0.6 (2)
O1—C4—C5—C6	−108.93 (16)	C17—C16—C21—C20	4.1 (2)
C15—C4—C5—C6	−10.8 (2)	C13—C16—C21—C20	−177.64 (15)
C3—C4—C5—C6	143.28 (15)	C20—C19—C22—C23	−176.47 (17)
C12—O2—C6—C5	135.46 (13)	C18—C19—C22—C23	3.8 (3)
C12—O2—C6—C7	14.37 (17)	C19—C22—C23—C33	−4.2 (3)
O1—C5—C6—O2	44.08 (18)	C19—C22—C23—C24	177.52 (16)
C4—C5—C6—O2	116.99 (15)	C22—C23—C24—C29	159.30 (17)
O1—C5—C6—C7	161.85 (13)	C33—C23—C24—C29	−19.1 (2)
C4—C5—C6—C7	−125.24 (16)	C22—C23—C24—C25	−19.2 (3)
O2—C6—C7—C11	−11.56 (16)	C33—C23—C24—C25	162.36 (15)
C5—C6—C7—C11	−130.62 (13)	C29—C24—C25—C26	−2.5 (2)
O2—C6—C7—C8	−135.79 (13)	C23—C24—C25—C26	175.96 (15)
C5—C6—C7—C8	105.15 (15)	C30—O4—C26—C25	−16.8 (2)
C11—C7—C8—C9	147.81 (13)	C30—O4—C26—C27	164.02 (14)
C6—C7—C8—C9	−94.58 (15)	C24—C25—C26—O4	−179.51 (15)
C7—C8—C9—C10	70.67 (17)	C24—C25—C26—C27	−0.4 (2)
C2—C1—C10—C14	−8.4 (3)	C31—O5—C27—C28	−63.9 (2)
C2—C1—C10—C9	171.04 (14)	C31—O5—C27—C26	119.53 (16)
C8—C9—C10—C1	−104.35 (17)	O4—C26—C27—O5	−0.7 (2)
C8—C9—C10—C14	75.17 (18)	C25—C26—C27—O5	−179.93 (14)
C8—C7—C11—C13	−54.0 (2)	O4—C26—C27—C28	−177.44 (14)
C6—C7—C11—C13	−178.52 (17)	C25—C26—C27—C28	3.4 (2)
C8—C7—C11—C12	130.10 (14)	C32—O6—C28—C29	−2.5 (2)
C6—C7—C11—C12	5.60 (16)	C32—O6—C28—C27	177.21 (15)
C6—O2—C12—O3	172.42 (15)	O5—C27—C28—O6	0.3 (2)
C6—O2—C12—C11	−10.86 (17)	C26—C27—C28—O6	176.89 (14)
C13—C11—C12—O3	2.6 (3)	O5—C27—C28—C29	179.97 (15)
C7—C11—C12—O3	179.18 (17)	C26—C27—C28—C29	−3.4 (2)
C13—C11—C12—O2	−173.76 (14)	C25—C24—C29—C28	2.5 (2)
C7—C11—C12—O2	2.78 (18)	C23—C24—C29—C28	−176.03 (15)
C12—C11—C13—C16	170.76 (16)	O6—C28—C29—C24	−179.82 (16)
C7—C11—C13—C16	−4.8 (3)	C27—C28—C29—C24	0.5 (2)
