# Supplementary figures and images for: Crystal structure of (E)-13-{4-[(Z)-2-cyano-2-(3,4,5-tri­meth­oxy­phen­yl)ethen­yl]phen­yl}parthenolide methanol hemisolvate
Source: Acta Crystallogr Sect E Struct Rep Online. 2014 Sep 6;70(Pt 10):o1092–3. doi: 10.1107/S1600536814019333 (PMC4257209; doi:10.1107/S1600536814019333)

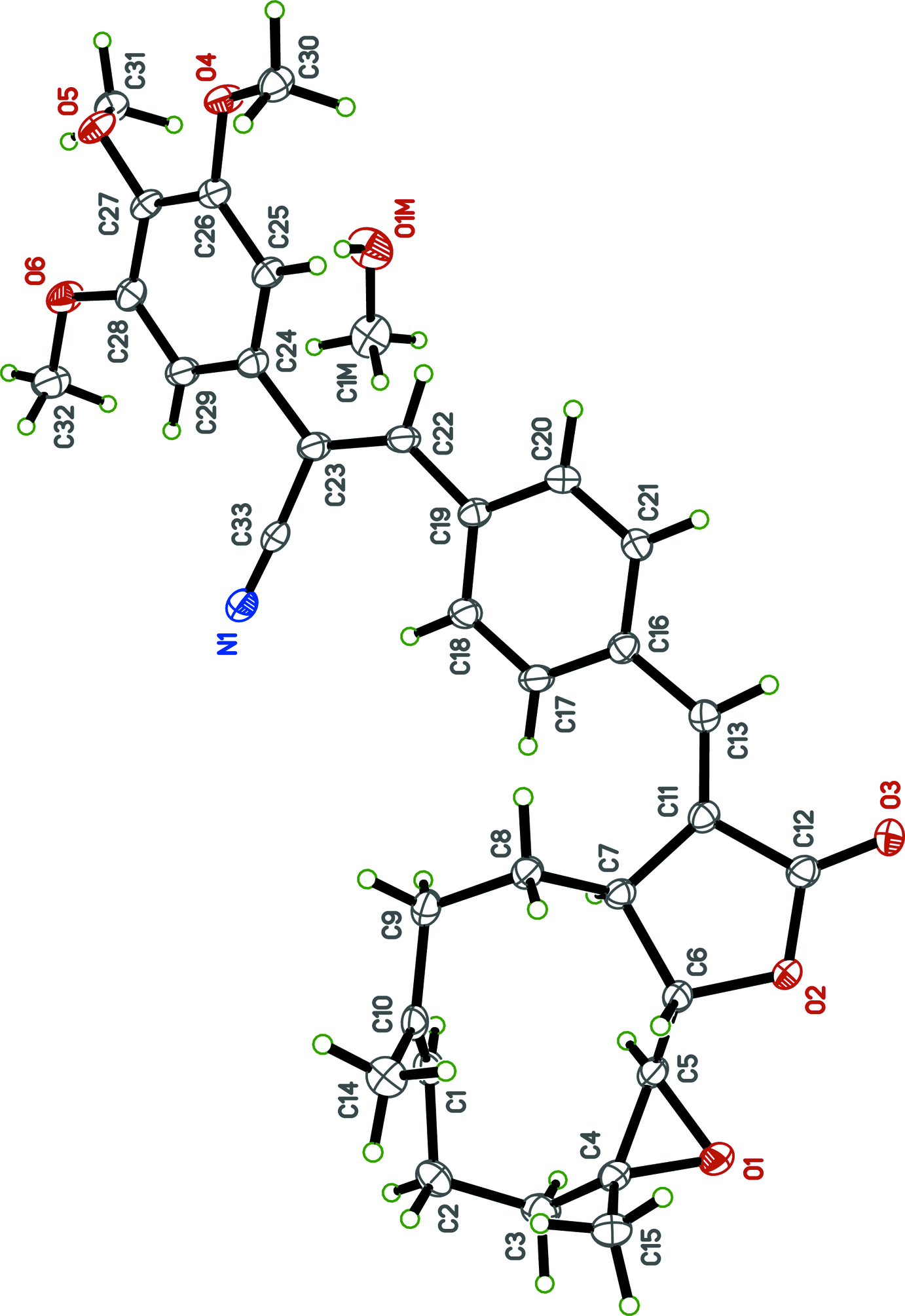

Supplement: Supplementary file 3 [file e-70-o1092-fig1.tif]
